# Prognostic significance of *FLT3*-ITD length in AML patients treated with intensive regimens

**DOI:** 10.1038/s41598-021-00050-x

**Published:** 2021-10-20

**Authors:** Tamara Castaño-Bonilla, Juan M. Alonso-Dominguez, Eva Barragán, Rebeca Rodríguez-Veiga, Claudia Sargas, Cristina Gil, Carmen Chillón, María B. Vidriales, Raimundo García, Joaquín Martínez-López, Rosa Ayala, María J. Larrayoz, Eduardo Anguita, Rebeca Cuello, Alberto Cantalapiedra, Estrella Carrillo, Elena Soria-Saldise, Jorge Labrador, Isabel Recio, Lorenzo Algarra, Carlos Rodríguez-Medina, Cristina Bilbao-Syeiro, Juan A. López-López, Josefina Serrano, Erik De Cabo, María J. Sayas, María T. Olave, Joaquín Sánchez-García, Mamen Mateos, Carlos Blas, Jose L. López-Lorenzo, Daniel Lainez-Gonzalez, Juana Serrano, David Martínez-Cuadrón, Miguel A. Sanz, Pau Montesinos

**Affiliations:** 1 Hematology Department, Hospital Universitario Fundación Jiménez Díaz, Avenida Reyes Católicos, 2, 28040 Madrid, Spain; 2 Instituto de Investigación Sanitaria (IIS-FJD), Hospital Universitario Fundación Jiménez Díaz, Madrid, Spain; 3 Hematology Department, Hospital Universitario La Fe de Valencia, Valencia, Spain; 4 Hematology Department, Hospital General de Alicante, Alicante, Spain; 5 Hematology Department, Hospital Universitario de Salamanca, Salamanca, Spain; 6 Hematology Department, Hospital General de Castellón, Castellón, Spain; 7 Hematology Department, Hospital Universitario Doce de Octubre, Complutense University, CNIO, Madrid, Spain; 8 Molecular Biology Department, Cimalab Diagnosis, Clínica Universitaria de Navarra, Navarra, Spain; 9 Hematology Department, Hospital Universitario Clínico San Carlos, Medicine Department, UCM, Madrid, Spain; 10 Hematology Department, Hospital Universitario de Valladolid, Valladolid, Spain; 11 Hematology Department, Hospital Universitario Río Hortega, Valladolid, Spain; 12 Hematology Department, Hospital Universitario Virgen del Rocío, Instituto de Biomedicina de Sevilla (IBIS/CISC/CIBERON), Sevilla, Spain; 13 Hematology Department, Hospital Universitario de Burgos, Burgos, Spain; 14 Hematology Department, Hospital Ntra. Sra. de Sonsoles de Ávila-Complejo Asistencial Ávila, Ávila, Spain; 15 Hematology Department, Hospital General de Albacete, Albacete, Spain; 16 Hematology Department, Hospital Universitario de Gran Canaria Doctor Negrín, Las Palmas de Gran Canaria, Spain; 17 Hematology Department, Hospital General Ciudad de Jaén, Jaén, Spain; 18 UGC de Hematologia, Hospital U. Reina Sofia, IMIBIC, UCO, Cordoba, Córdoba, Spain; 19 Hematology Department, Hospital Comarcal del Bierzo, León, Spain; 20 Hematology Department, Hospital Universitario Doctor Peset, Valencia, Spain; 21 Hematology Department, Hospital Clínico Universitario Lozano Blesa, Zaragoza, Spain; 22 Hematology Department, Complejo Hospitalario de Navarra, Navarra, Spain

**Keywords:** Oncogenesis, Acute myeloid leukaemia, Prognostic markers, Molecular medicine

## Abstract

*FLT3-*ITD mutations are detected in approximately 25% of newly diagnosed adult acute myeloid leukemia (AML) patients and confer an adverse prognosis. The *FLT3*-ITD allelic ratio has clear prognostic value. Nevertheless, there are numerous manuscripts with contradictory results regarding the prognostic relevance of the length and insertion site (IS) of the *FLT3*-ITD fragment. We aimed to assess the prognostic impact of these variables on the complete remission (CR) rates, overall survival (OS) and relapse-free survival (RFS) of AML patients with *FLT3*-ITDmutations. We studied the *FLT3*-ITD length of 362 adult AML patients included in the PETHEMA AML registry. We tried to validate the thresholds of ITD length previously published (i.e., 39 bp and 70 bp) in intensively treated AML patients (n = 161). We also analyzed the mutational profile of 118 *FLT3*-ITD AML patients with an NGS panel of 39 genes and correlated mutational status with the length and IS of ITD. The AUC of the ROC curve of the ITD length for OS prediction was 0.504, and no differences were found when applying any of the thresholds for OS, RFS or CR rate. Only four out of 106 patients had ITD IS in the TKD1 domain. Our results, alongside previous publications, confirm that *FLT3*-ITD length lacks prognostic value and clinical applicability.

## Introduction

Acute myeloid leukemia (AML) is the most common form of acute leukemia in adults. The clinical behavior and genetic characteristics of the disease are heterogeneous^1^. In the last 25 years, advances in molecular techniques have allowed a greater understanding of the pathogenesis of AML and the subsequent development of targeted therapies and a more refined prognostic classification based on the genetic features of the disease^2,3^.

FMS-like tyrosine kinase-3 internal tandem duplication (*FLT3*-ITD) is one of the most frequent mutations found in AML patients. Mutations of *FLT3* are found in approximately 30% of newly diagnosed AML patients and appear either as ITDs (≈ 25%) or point mutations in the tyrosine kinase domain (TKD) (7–10%)^4^. *FLT3*-ITD mutations occur in the form of a replicated sequence in the juxtamembrane domain (JMD) and/or TKD1 of the *FLT3* gene. *FLT3*-ITD is located within exon 14, corresponding to JMD, in 70% of AML patients, while 30% of ITDs span exon 15, corresponding to the TKD1 domain. *FLT3*-ITDs show great variation in size (ranging from 3 to more than 400 base pairs (bp)), insertion sites (ISs), allelic ratios (ARs) and the number of clones^5^.

AML patients with *FLT3*-ITD mutations show an increased relapse rate, reduced disease-free survival (DFS), and decreased long-term survival, while the rate of complete remission (CR) after induction chemotherapy is not significantly affected^6,7^. *FLT3*-ITD mutational load, expressed as an AR determined by fragment length analysis, has a clear prognostic value and is, therefore, included in the genetic prognostic classification of the European Leukemia Net (ELN) published in 2017^8^. Nevertheless, there are numerous and contradictory manuscripts regarding the prognostic importance of the length and insertion site of the ITD fragment. Some studies showed a reduced CR rate, while others analyzing the IS in the same region found differences in OS. Additionally, different subdomains have been highlighted, such as those conferring an adverse outcome^9,10^. Regarding ITD length, some authors have found that patients with shorter ITD lengths have more favorable outcomes^11,12^ or worse prognoses^13^, while other researchers did not find a prognostic relationship^14^. None of the studies has carried out an internal ITD length cutoff validation by dividing the patients into a training cohort and a validation cohort, which, given the arbitrary selection of the cutoffs used, would be necessary. Nevertheless, some thresholds have been applied in more than one study [i.e., 39 bp and 70 bp]^11,15–17^. We aimed to shed light on the prognostic importance of the *FLT3*-ITD length and site of insertion by validating previously suggested sites of insertion and thresholds of ITD length. Moreover, we performed an analysis of the correlation of *FLT3-*ITD length and insertion sites with the mutational landscape of AML, which has not been carried out thus far.

## Results

### Patient characteristics and treatment

Intensive chemotherapy regimens were administered to 161 patients (idarubicin + cytarabine; 3 + 7, n = 151 and 2 + 5, n = 8; IDA-FLAG (fludarabine + Ara-C + idarubicin), n = 1 and FLAG, n = 1). The median age of this group was 55.1 years (range 17.1–85.3 years); 76 males and 85 females. The non-intensive chemotherapy group received FLUGA (fludarabine + Ara-C), n = 22; azacytidine, n = 15; and decitabine, n = 5, and one patient was treated with IDA-FLAG-Lite. Regardless of the regimen intensity, all clinical trial participants were grouped in a separate treatment category (n = 15). The BSC group included 7 patients receiving transfusions and other supportive measures. As consolidation therapy, one hundred patients received high-intensity treatment (3 + 7, n = 68; 3 + 7 + gemtuzumab ozogamicin (GO), n = 4; 2 + 5 = 2; IDA-FLAG, n = 1; high-dose cytarabine (HDARAC), n = 23; low-dose cytarabine (LDARAC), n = 1; and Ara-C 100 mg/m2 × 5, n = 1). Seventeen patients underwent autologous hematopoietic progenitor transplantation, and forty-four patients underwent allogeneic hematopoietic progenitor transplantation (Table 1).

**Table 1:** Baseline characteristics of *FLT3*-ITD AML patients treated with intensive chemotherapy regimens.

Patient characteristics	Total N = 161	ITD lentgh < 39 bp N = 48	ITD lentgh ≥ 39 bp N = 113	ITD lentgh < 70 bp N = 119	ITD lentgh ≥ 70 bp N = 42
Median age at diagnosis, years	55.1	55.7	52.4	55.1	52.3
Range	17–85	17–85	17–74	17–85	20–74
Median follow-up, years	0.8	0.8	0.8	0.8	0.9
Range	0–12.5	0–10.2	0–12.5	0–12.5	0–6.4
**Sex**					
Female	85 (52.8)	23 (47.9)	62 (54.9)	60 (50.4)	25 (59.5)
Male	76 (47.2)	25 (52.1)	51 (45.1)	59 (49.6)	17 (40.5)
Median ITD allelic ratio (AR)	0.6	0.6	0.6	0.6	0.6
**ITD allelic ratio ELN 2017**					
AR < 0.5	58 (36.0)	17 (35.4)	41 (36.3)	43 (36.1)	15 (35,7)
AR ≥ 0.5	82 (51.9)	23 (47.9)	59 (52.2)	61 (51.2)	21 (50,0)
**FAB subtypes**					
M0	5 (3.1)	1 (2.1)	4 (3.5)	3 (2.5)	2 (4.7)
M1	36 (22.4)	11 (22.9)	25 (22.1)	26 (21.8)	10 (23.8)
M2	12 (7.5)	5 (10.4)	7 (6.2)	10 (8.4)	2 (4.8)
M4	22 (13.7)	10 (20.8)	12 (10.6)	16 (13.4)	6 (14.3)
M5	33 (20.5)	8 (16.6)	25 (22.1)	27 (22.7)	6 (14.3)
M6	1 (0.6)	1 (2.1)	─	1 (0.8)	─
M7	1 (0.6)	─	1 (0.6)	1 (0.8)	─
Unclassified	6 (3.7)	─	6 (5.3)	4 (3.4)	2 (4.8)
**Cytogenetic risk (ELN 2010)**					
Favorable risk	─	─	─	─	─
Intermediate-I risk	99 (61.4)	31 (64.6)	68 (60.1)	75 (63.0)	24 (57.1)
Intermediate-II risk	15 (9.3)	5 (10,4)	10 (8.8)	14 (11.8)	1 (2.4)
Adverse risk	9 (5.6)	2 (4,1)	7 (6.2)	6 (5.0)	3 (7.1)
Median leucocytes at diagnosis, 109/L	50.7	49.6	54.0	50.7	55.5
Range	0.6–434	0.8–434.3	0.6–371.8	0.6–434.3	0.8–365.5
Median hemoglobin at diagnosis, g/dl	8.9	8.9	8.8	8.9	8.7
Range	4–15.6	4–14.7	4–15.6	4–14.7	6.4–15.6
Median platelets at diagnosis, 109/L	48	47	47	48	46.5
Range	9–330	17–330	9–246	9–330	10.7–217
**Induction therapy**					
Idarubicin + Cytarabine (3 + 7)	150 (92.5)	42 (85.4)	108 (95.5)	110 (91.6)	40 (95.2)
Idarubicin + Cytarabine (2 + 5)	8 (5.0)	4 (8.3)	4 (3.5)	6 (5.0)	2 (4.8)
Daunorubicin + Cytarabine (3 + 7)	1 (0.6)	1 (2.1)	─	1 (0.8)	─
IDA-FLAG	1 (0.6)	─	1 (0.9)	1 (0.8)	─
FLAG	1 (0.6)	1 (2.1)	─	1 (0.8)	─
**Stem cell transplant**					
Allogeneic hematopoietic cell transplantation	44 (27.3)	15 (31.3)	29 (25.6)	33 (27.7)	11 (26.2)
Autologous hematopoietic cell transplantation	17 (10.6)	4 (8.3)	13 (11.5)	12 (10.1)	5 (11.9)

*FAB* French American-British, *ELN* European leukemia net.

### Impact of the FLT3-ITD allelic ratio

The *FLT3*-ITD AR was available in 140 intensively treated patients. We used the 0.5 cutoff of the AR as recommended by the 2017 ELN guidelines^8^.These patients were divided on the basis of the *FLT3*-ITD AR into an *FLT3*-ITD^LOW^group (41%; n = 58) and an *FLT3*-ITD^HIGH^group (59%; n = 82). In the *FLT3*-ITD^LOW^ group of patients, the median OS was 2.3 years (CI: 1.1–3.6), and in the *FLT3*-ITD^HIGH^ group of patients, the median OS was 1.1 years (CI: 0.7–1.5). When comparing both subgroups using a log-rank test, there was a clear trend toward a reduced OS in *FLT3*-ITD^HIGH^ patients (*P* = 0.052).

### Length of FLT3-ITD mutations

The length of the 362 ITDs ranged from 3 to 201 bp, with a median ITD length of 48 bp.The distribution of ITD length can be observed in Supplementary Fig. S1. In those patients with more than one ITD mutation, only the longest mutation was selected for statistical analysis (10 patients had > 1 ITD mutation). The area under the ROC curve (AUC) for OS prediction was 0.504. We also performed an ROC curve analysis for OS prediction excluding those 10 patients with more than 1 ITD insertion and obtained an AUC of 0.521. As we have already explained, our main goal was to validate two previous recurrently applied cutoffs: 39 bp and 70 bp. Nevertheless, we also performed an analysis with the median ITD length of our cohort (48 bp). The analysis of OS and RFS applying this value did not show significant results (data not shown).

#### Impact of FLT3-ITD size using the 39 bp cutoff

**Figure 1:**
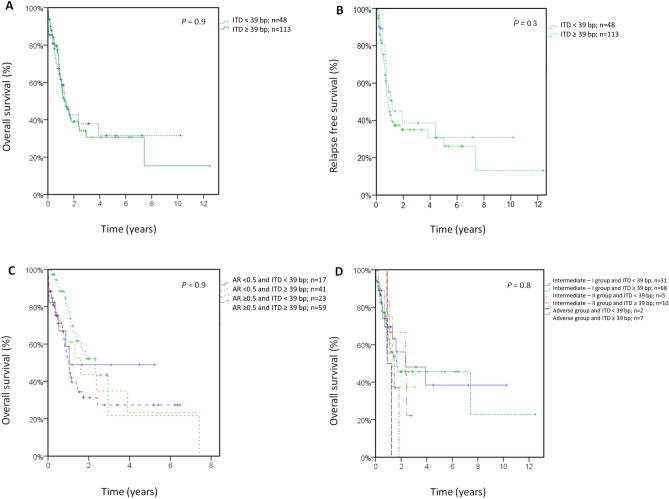
Clinical outcome stratified according to the *FLT3*-ITD length (cutoff 39 bp) for all patients treated with intensive chemotherapy. (**A**) Overall survival. (**B**) Relapse-free survival. (**C**) OS according to the *FLT3*-ITD length and allelic ratio. (**D**) OS according to the *FLT3*-ITD length and 2010 ELN genetic risk. AR,allelic ratio.

First, 161 AML patients with *FLT3*-ITD mutations treated with IC were analyzed using 39 bp as the cutoff (< 39 bp; n = 48, ≥ 39 bp; n = 113). The median OS was 1.3 years (CI: 0.7–1.9) and 1.4 years (CI: 0.9–1.9), respectively (*P* = 0.9). The median RFS was 1.2 years (CI: 0–2.4) and 0.9 years (CI: 0.6–17.1), respectively (*P* = 0.3). CR or CRi was achieved in 70% of the patients in both groups (*P* = 0.9). An analysis of OS censoring at the time of allo-HSCT did not yield significant results (data not shown).A stratified analysis of *FLT3*-ITD length on the basis of the AR was performed in 140 patients (AR < 0.5 and ITD < 39 bp, n = 17; AR < 0.5 and ITD ≥ 39 bp, n = 41; AR > 0.5 and ITD < 39 bp, n = 23; AR > 0.5 and ITD ≥ 39 bp, n = 59). The median OS was 1.0 years [CI not calculable (NC)], 2.3 years (CI: 1.2–3.5), 1.6 years (CI: 0.6–2.6) and 1.0 years (CI: 0.8–1.2), respectively (*P* = 0.9). Similarly, a stratified analysis of *FLT3*-ITD length on the basis of 2010 ELN genetic risk was performed in 123 patients (intermediate-I group, ITD < 39 bp, n = 31 and ITD ≥ 39 bp, n = 68; intermediate-II group, ITD < 39 bp, n = 5 and ITD ≥ 39 bp, n = 10; and adverse group, ITD < 39 bp, n = 2 and ITD ≥ 39 bp, n = 7). The median OS was 2.4 years (CI 0–5.5), 1.7 years (CI: 0–4.4), 1.3 years (CI 0.6–2.0), 1.5 years (CI: 0.2–2.7), 0.9 years (CI NC) and 2.3 years (CI: 0–4.8), respectively. No statistically significant differences were found (*P* = 0.8) (Fig. 1).

#### Impact of FLT3-ITD size using the 70 bp cutoff

**Figure 2:**
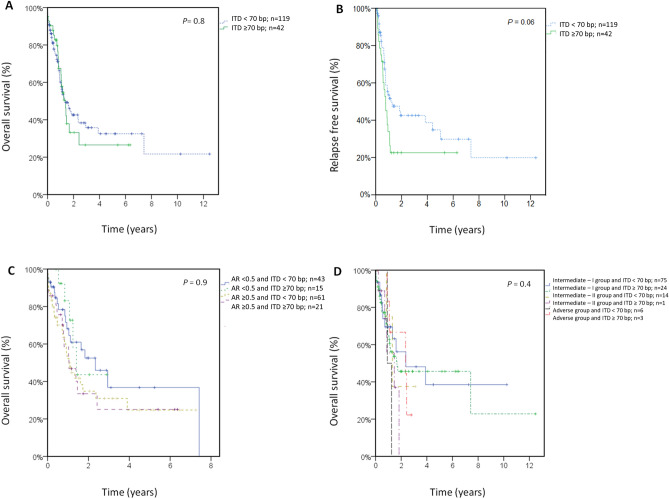
Clinical outcome stratified according to the *FLT3*-ITD length (cutoff 70 bp) for all patients treated with intensive chemotherapy. (**A**) Overall survival. (**B**) Relapse-free survival. (**C**) OS according to the *FLT3*-ITD length and allelic ratio. (**D**) OS according to the *FLT3*-ITD length and 2010 ELN genetic risk. AR, allelic ratio.

Prognostic analyses were performed using the 70 bp cutoff in 161 AML patients with *FLT3*-ITD mutations treated with IC (< 70 bp; n = 119, ≥ 70 bp; n = 42). The median OS was 1.3 years (CI 0.7–1.9) and 1.4 years (CI 1.0–1.8), respectively (*P* = 0.8). The median RFS was 1.2 years (CI 0.2–2.2) and 0.77 years (CI 0.5–1.1), respectively (*P* = 0.06). CR or CRi was achieved in 70% of the patients in both groups (*P* = 0.9). An analysis of OS censoring at the time of allo-HSCT did not yield significant results (data not shown). A stratified analysis of *FLT3*-ITD length on the basis of the AR was performed in 140 patients (AR < 0.5 and ITD < 70 bp, n = 43; AR < 0.5 and ITD ≥ 70 bp, n = 15; AR > 0.5 and ITD < 70 bp, n = 61; AR > 0.5 and ITD ≥ 70 bp, n = 21). The median OS was 2.3 years (CI: 1.0–3.6), 1.4 years (CI: 1.0–1.8), 1.1 years (CI: 0.8–1.3) and 1.0 years (CI: 0.3–1.8), respectively (*P* = 0.9). A stratified analysis of *FLT3*-ITD length by 2010 ELN genetic risk was performed in 123 patients (intermediate-I group, ITD < 70 bp, n = 75 and ITD ≥ 70 bp, n = 24; intermediate-II group, ITD < 70 bp, n = 14 and ITD ≥ 70 bp, n = 1; and adverse group, ITD < 70 bp, n = 6 and ITD ≥ 70 bp, n = 3). The median OS was 1.7 years (CI 0–4.0), 1.7 years (CI NC), 1.3 years (CI 0.3–2.3), 1.5 years (CI NC), 1.2 years (CI: 0.5–2.0) and 2.4 years (CI NC), respectively. No statistically significant differences were found (*P* = 0.4) (Fig. 2).

There were two patients with core binding factor (CBF) translocations (one *RUNX-RUNX1T1* and one *CBFB-MYH11*) and *FLT3*-ITD mutations. CBF translocations have been associated with *FLT3*-ITD mutations in very few patients, and there is no clear information regarding their ELN prognostication^18–20^. Therefore, these patients were not included in the analysis stratified by 2010 ELN genetic risk^21^.

### Insertion site of the FLT3-ITD mutations

**Figure 3:**
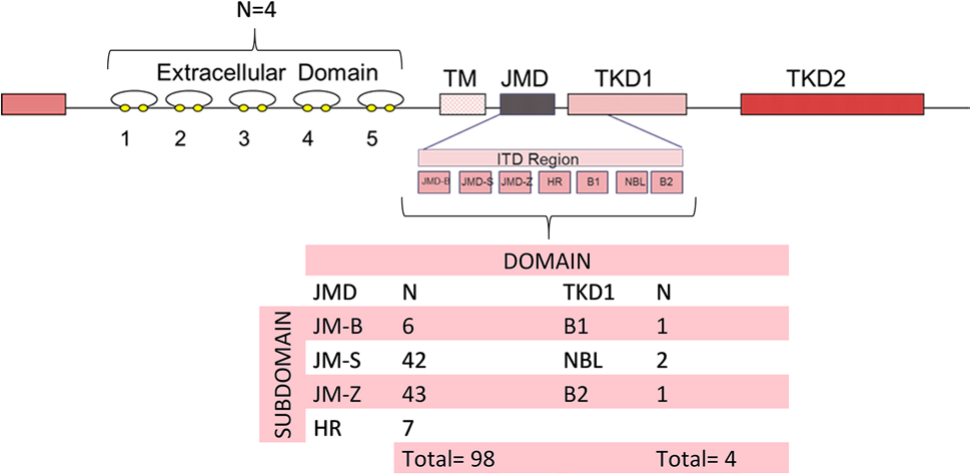
Analysis of *FLT3*-ITD insertion sites from 106 *FLT3*-ITD-positive AML patients. In our cohort, *FLT3*-ITD was located in the JMD domain (JMD-B, JMD-S, JMD-Z and HR) in 98 patients and in the TKD1 domain (B1, NBL and B2) in four patients. A detailed analysis of all patients showed ITD integrations in the JMD-B, amino acids 572 to 578, in six patients; the JMD-S, amino acids 579 to 592, in 42 patients; the JMD-Z, amino acids 593 to 603, in 43 patients; the HR, amino acids 604 to 609, in seven patients; the B1 of TKD1, amino acids 610 to 615, in one patient; the NBL, amino acids 616 to 623, in two patients; and the B2, amino acids 624 to 630, in one patient. TM,transmembrane domain; JMD, juxtamembrane domain; JMD-B, binding motif; JMD-S, switch motif; JMD-Z, zipper motif; HR, hinge region; TKD1, tyrosine kinase domain 1; B1, beta1-sheet; NBL, nucleotide binding loop; B2, beta2-sheet; and TKD2, tyrosine kinase domain 2.

The insertion site was analyzed in 106 AML patients with the *FLT3*-ITD mutation. Regrettably, patients with information on the IS of ITD available had received different treatments: intensive chemotherapy, n = 37; non-intensive therapy, n = 14; clinical trials, n = 6; and best supportive care, n = 2. We have no information on the treatment received by the remaining patients. Ninety-eight patients had ITD insertion sites in the JMD domain (JM-B, n = 6; JM-S, n = 42; JM-Z, n = 43; and hinge region (HR), n = 7), four patients had ITD insertion sites in the TKD1 domain (beta1-sheet, n = 1; beta2-sheet, n = 1; and nucleotide binding loop (NBL), n = 2) and four patients had ITD insertion sites in the extracellular domain (ED) (Fig. 3). Therefore, only 3.8% of the patients showed an *FLT3*-ITD insertion in the TKD1 domain. Given the heterogeneity of treatments received and the scarce number of ISs in TKD1, we did not perform statistical analysis.

### Correlation between FLT3-ITD length or insertion site and other gene variants by NGS

**Figure 4:**
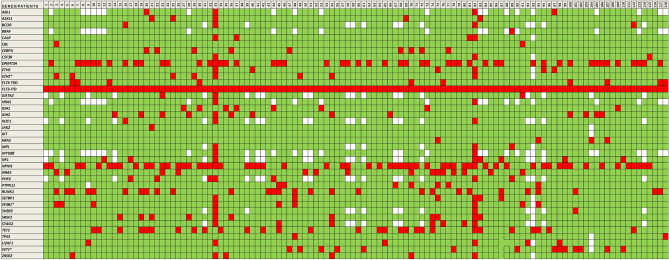
The landscape of mutations identified by NGS in AML patients. These mutations arearranged in increasing order by *FLT3*-ITD length. Green indicates non-mutated genes, red indicates mutated genes and white indicates non-mutated genes. * Genes with a *P* value < 0.05 in the Mann–Whitney test correlating mutational status with ITD length.

The median length of the ITD in four patients with *SF3B1 *mutations was 15 bp vs 48 bp in patients without *SF3B1* mutations (n = 64) (*P* = 0.012). Similarly, the median ITD length in three patients with *EZH2 *mutations was 26 bp vs 48 bp in the wild-type group (n = 65) (*P* = 0.031). Furthermore, ten patients with mutated *WT1* showed a median ITD length of 77 bp, and 58 patients with non-mutated *WT1* showed a median ITD length of 42 bp (*P* = 0.021). Mutations of *SF3B1*, *EZH2* and *WT1* seem to be a more ancestral event than *FLT3* mutations, as expected, given the VAF of the genes. Nevertheless, in three patients, similar VAFs (< 5% difference) were detected, which might indicate that these mutations occurred at the same timepoint as the *FLT3* mutation.No significant differences were found between the ITD length and the mutational status of any of the remaining genes (Fig. 4).

All four patients with ITD insertions in TKD1 had mutations in *DNTM3A*, compared with 39 out of 96 patients (41%) with ITD insertions in the JMD domain (*P* = 0.031). Statistically significant results were not observed for any other gene in this analysis.

## Discussion

This study shows that the size of *FLT3*-ITD mutations has no prognostic impact in terms of survival, relapse or CR rate among newly diagnosed AML patients treated with first-line intensive regimens. Our real-life cohort was composed of 362 patients, most of whom were not included in clinical trials. As in previous works, we analyzed the clinical significance of *FLT3*-ITD length among fit patients treated with intensive regimens^15,16^. Our median ITD length was 48 bp (range = 3 bp to 231 bp), similar to previous studies^12,14,17^. To test the prognostic significance of the ITD length and its clinical applicability, we used recurrent previously published cutoffs, which were analyzed in series ranging from 28 to 100 intensively treated patients. Patients with an ITD fragment ≥ 39 bp or ≥ 70 bp had a significant reduction in OS and RFS in some of these studies, but we were unable to validate these findings^11,15–17^. We obtained a *P* value of 0.055 in the analysis of RFS applying the 70 bp cutoff. Of note, we tested 3 different ITD length thresholds, and to be considered significant, the *P* value should be < 0.025. Therefore, the value obtained is not significant, although it shows a slight trend toward being significant. The size of our cohort was larger than those of the studies published using these cutoffs. Larger studies of ITD size, although they did not employ these cutoffs, did not find prognostic power of this measure, which corroborates our results. Additionally, the area under the ROC curve, which serves as an indicator of the diagnostic capacity of the ITD length as a whole, was 0.504. This value highlights the scarce prognostic value of the measure. On the other hand, we obtained a value (0.52) that was close to significant in the analysis of the prognostic impact of the *FLT3-*ITD AR according to the 2017 ELN cutoff^8^.

Regarding the ITD insertion site, Kayser et al^22,23^ observed that adult AML patients with an ITD in the beta-1 sheet had significantly inferior OS and DFS compared to those with ITDs located in other regions. However, other studies did not find significantly worse clinical outcomes in patients with non-JMD ITD mutations^24,25^. Finally, a different report showed worse clinical outcomes in terms of OS and DFS in the TKD1 group. Additionally, different subdomains of TKD1 (HR or beta1-sheet) have been highlighted as those conferring an adverse outcome^10^. Therefore, there is a lack of consensus regarding the prognostic importance of the ITD IS and the subdomains that confer this adverse outcome. Unfortunately, in our study, information on the site of insertion was not available in the whole cohort, and few patients harbored a TKD1 insertion.We did not carry out a statistical analysis of the insertion site given the heterogeneity in the treatment of patients analyzed and the small number of patients with an ITD inserted in the TKD1 domain. We have no explanation regarding the reduced number of patients with an *FLT3*-ITD inserted in TKD1 found in our cohort.

Given the increasing importance that massive sequencing techniques are acquiring in the prognosis determination and therapeutic management of AML patients, we decided to study the possible correlation between the length or site of the insertion of the mutated ITD fragment and the mutational profile of these patients. We found a statistically significant correlation among *SF3B1*, *WT1* and *EZH2* mutations and ITD length. Interestingly, all patients with an *FLT3*-ITD inserted in the TKD1 domain showed *DNMT3A* mutations. Taking into account the great number of comparisons performed, we cannot assume a real relationship between these mutations. More studies will be necessary to confirm these results and to shed light on the possible physiopathologic relationship.

Our study has several limitations: (1) Our patients were selected from an observational registry, which can be interpreted as a limitation given the heterogeneity of treatments or as a strength because our data are thereby more similar to those observed in real-life clinical practice than those derived from a clinical trial^26,27^. (2) Larger studies analyzing ITD length also found no significant results^14,23,28^. However, these studies did not apply previously validated ITD length cutoffs obtained in other smaller series^11,15–17^. (3) Findings regarding the relationship between ITD length/site and mutational profile might be interpreted as exploratory given the high number of correlations performed. (4) Only five patients in our cohort received treatment with midostaurin (2 in induction and 3 in consolidation treatment); therefore, we were not able to draw conclusions regarding the prognostic impact of the length of the ITD as described in previous studies^29,30^. The impact of prognostic factors may change as the AML treatment landscape evolves. (5) No data regarding minimal residual disease (MRD) were available in our cohort, and MRD data could be interesting to analyze in future studies.

We suggest that any investigator who wants to demonstrate the prognostic value of the ITD length applies some of the recurrent published thresholds used in this study or divides his cohort into training and validation subcohorts. The data described in the literature alongside the results that we have obtained regarding ITD mutation lead us to believe that future studies should focus on the functional characterization of the protein products of the mutated genes. Protein alteration seems to be much more complex than the length of the mutation or the site of insertion; therefore, our efforts to simplify *FLT3*-ITD characteristicsby stratifying the risk of the patients may be fruitless.

In summary, in our population of 161 intensively treated *FLT3*-ITD AML patients, we did not validate any of the previously published recurrent thresholds of ITD length obtained from smaller series. Our results, alongside those of other non-significant reports, lead us to believe that *FLT3*-ITD length has neither prognostic value nor possible clinical application.

## Material and methods

### Patients and samples

The Programa Español de Tratamientos en Hematología (PETHEMA) AML epidemiologic registry (NCT02607059) includes patients diagnosed with AML, regardless of the treatment administered. The main patient and disease characteristics were collected retrospectively, including demographic characteristics (age, sex), cytomorphologic assessments confirming the AML diagnosis (according to routine site practice), cytogenetics, molecular studies, first-line treatment approach, disease response assessment and disease follow-up. Patients diagnosed with acute promyelocytic leukemia (APL) were excluded. Among 729 AML patients with *FLT3*-ITD mutations included in the PETHEMA AML epidemiologic registry between 2003 and 2019, *FLT3*-ITD length was available in 362: 188 males and 174 females; median age of 60.8 years (range 17.1–91.4 years). Patients were classified into four therapeutic groups according to the first-line approach: intensive chemotherapy (IC), n = 161; non-intensive therapy, n = 43; clinical trial, n = 15; and best supportive care (BSC) only, n = 7.

**Figure 5:**
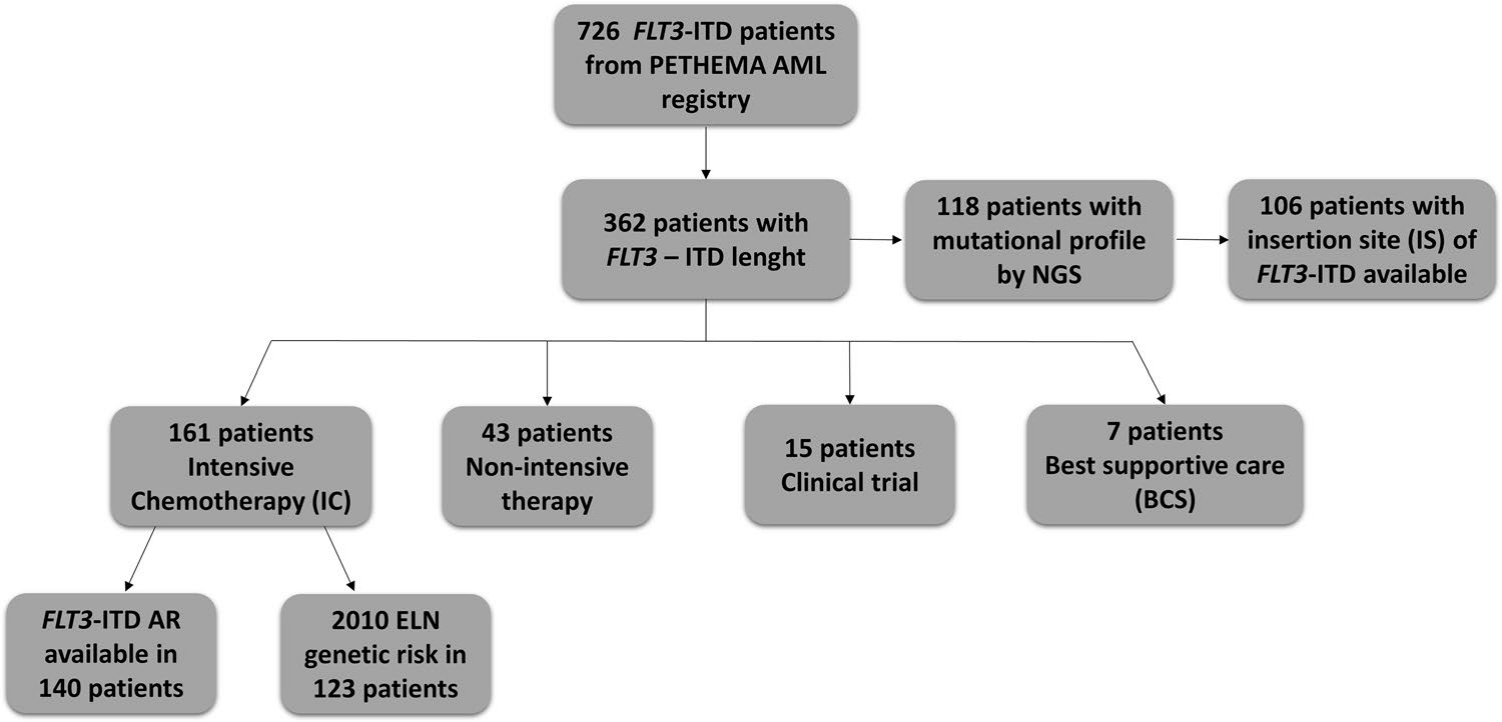
Flow diagram showing all AML patients with *FLT3-*ITD mutations in the study period between 2003 and 2019 on the basis of genetic data and treatment administered. NGS, next-generation sequencing.

Among 161 intensively treated patients, 123 had the cytogenetic and molecular information required to calculate the 2010 ELN classification^21^. Prognostic impact analyses of *FLT3*-ITD length were performed among patients treated with upfront IC regimens. Furthermore, a global query was sent to the different centralized laboratories of PETHEMA to verify the ITD length, insertion site and molecular profile of the patients by NGS when these data were available. Among 362 patients, NGS was performed in 118 patients using a panel of 39 genes. The insertion site of *FLT3*-ITD was available in 106 of 118 patients (Fig. 5).The study protocol was conducted following the guidelines of the Declaration of Helsinki and approved by the Ethics Committee for Clinical Research of the Hospital UniversitarioFundación Jiménez Díaz (PIC169-18_FJD). Informed consent was a requisite for patients alive at the time of data lock (January 2019).

### FLT3-ITD testing by PCR and capillary electrophoresis

*FLT3*-ITD fragment length analysis was performed in seven centralized PETHEMA laboratories. All samples investigated in this study were obtained at the time of diagnosis. DNA was extracted using automated or manual DNA extraction kits following the manufacturer’s recommendations. DNA quantification was performed with a Nanodrop (Thermo Fisher Scientific, Waltham,MA) or Qubitfluorometer (Thermo Fisher Scientific, Waltham, MA). PCR with fluorescently labeled primers followed by capillary electrophoresis for *FLT3*-ITD was performed as described elsewhere^31^. ITD amplicons with a size greater than that of the wild type (328 ± 1 bases) were interpreted as positive for the *FLT3*-ITD mutation. The number, area and length of mutant peaks on capillary electrophoresis were analyzed using GeneMapper analysis software (Applied Biosystems, Foster City, CA). The AR was determined by fragment length analysis and calculated as previously described^32^.

### Targeted next-generation sequencing

Samples from 118 of the 362 AML patients with *FLT3*-ITD mutations were analyzed with an NGS panel of 39 genes (see Supplementary Fig. S2) in PETHEMA centralized diagnostic laboratories as previously described^33^. Information regarding the ITD insertion site and mutational status of another 38 genes recurrently mutated in myeloid neoplasms was available in 106 and 118 patients, respectively.

### Statistical analysis

Overall survival (OS) was calculated from the date of the diagnosis of AML until death in all included patients. Relapse-free survival (RFS) was calculated from the date of achieving CR/CRi until the date of relapse (death without relapse or relapse were consideredevents)^8^. Complete response (CR) or complete responses with incomplete hematologic recovery (CRi) were defined according to current 2017 ELN guidelines^8^. Previously published cutoffs of ITD length, reported in more than one publication (i.e., 39 bp and 70 bp), were tested to check their applicability in our cohort. Kaplan–Meier analysis and log-rank tests were employed to compare different groups.We also carried out an additional OS analysis censoring patients at the time of allo-HSCT. Stratified Kaplan–Meier analysis was also employed with the AR and genetic risk, following 2010 ELN guidelines^21^, as classifiers of the patients. CR + CRi rates between groups were compared with a chi-square test. *FLT3*-ITD length was compared between mutation and wild-type groups for each of the 39 genes using a Mann–Whitney test. Fisher’s exact test was employed to correlate the ITD insertion site and mutational status. Statistical analyses were performed with SPSS 19.0 (IBM, Armonk, NY).

### Ethical approval

The study protocol was conducted following the guidelines of the Declaration of Helsinki and approved by the Ethics Committee for Clinical Research of the Hospital UniversitarioFundación Jiménez Díaz (PIC169-18_FJD).

### Informed consent

Informed consent was a requisite for patients alive at the time of data lock (January 2019).

## Supplementary Information

Supplementary Information.
