# Supplementary material for: Prognostic significance of FLT3-ITD length in AML patients treated with intensive regimens
Source: Sci Rep. 2021 Oct 20;11:20745. doi: 10.1038/s41598-021-00050-x (PMC8528825; doi:10.1038/s41598-021-00050-x)
Supplement: Supplementary file 1 — Supplementary Information. [file 41598_2021_50_MOESM1_ESM.docx]

**Supplementary material**


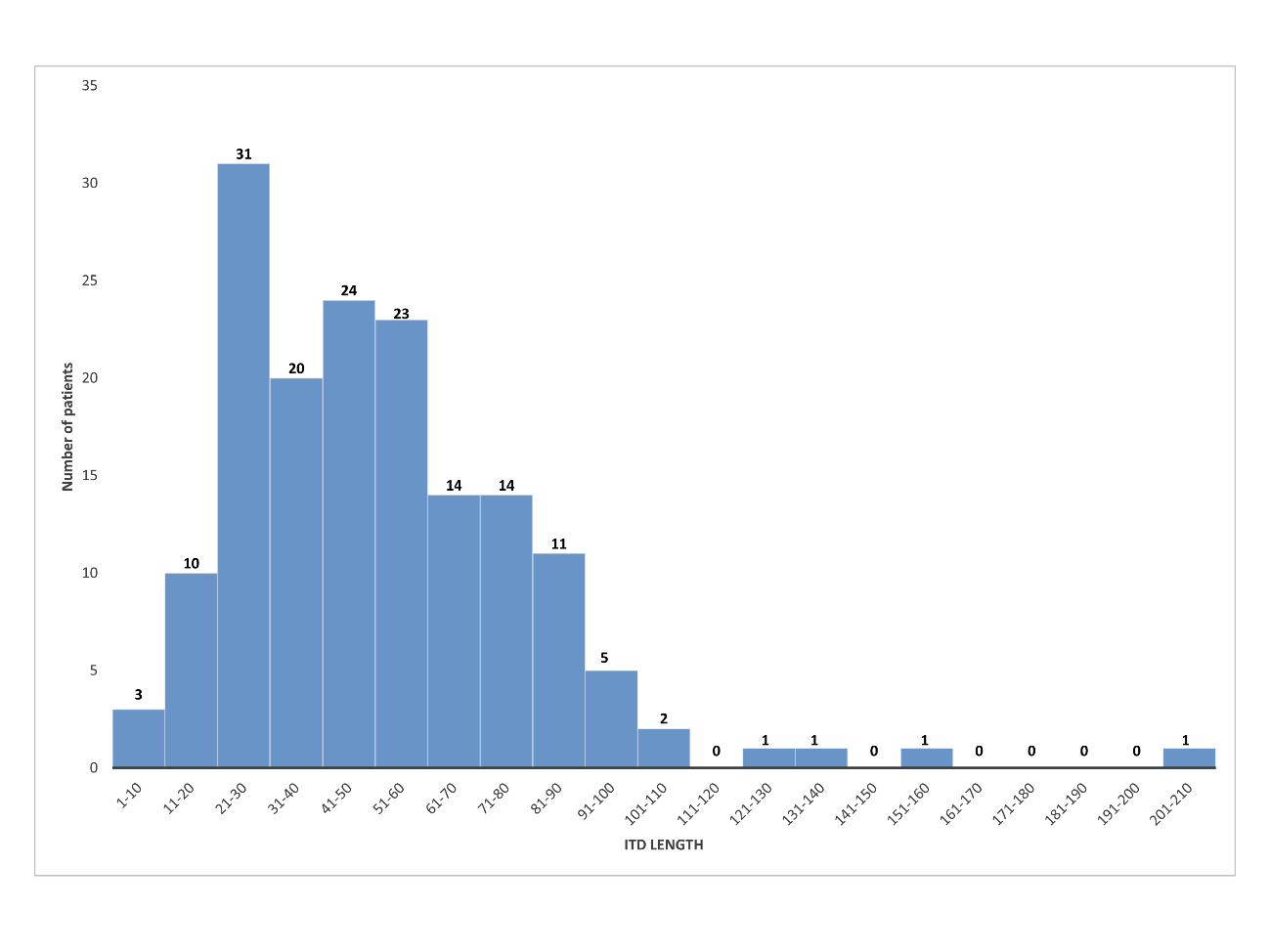


**Supplementary Fig. S1.** Distribution of ITD length in AML patients treated with intensive chemotherapy regimens


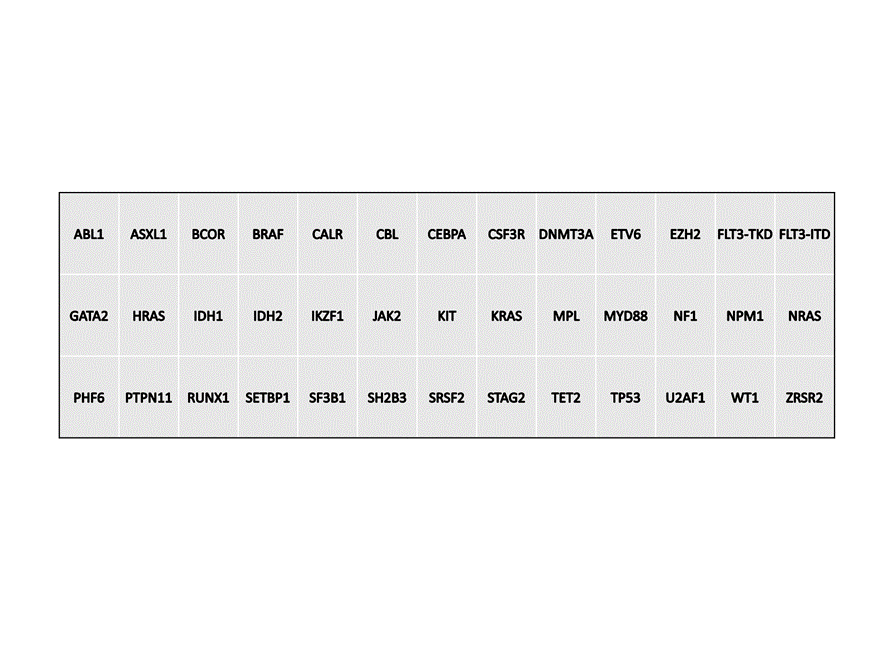


**Suplementary Fig. S2.** Panel of 39 genes used in the NGS study in 118 AML patients
